# Bridging the gap: a pragmatic approach to planetary health

**DOI:** 10.3389/fpubh.2024.1491457

**Published:** 2024-11-27

**Authors:** Rodrigo C. Menezes, Isabela B. B. Ferreira, Leonardo Martinez, Bruno B. Andrade

**Affiliations:** ^1^Planetary Health and Science Ensemble (PHASE) Consortium, Multinational Organization Network Sponsoring Translational and Epidemiological Research (MONSTER) Initiative, Salvador, Bahia, Brazil; ^2^Laboratório de Pesquisa Clínica e Translacional, Instituto Gonçalo Moniz, Fundação Oswaldo Cruz, Salvador, Bahia, Brazil; ^3^Instituto de Pesquisa Clínica e Translacional (IPCT), Faculdade Zarns, Clariens Educação, Salvador, Bahia, Brazil; ^4^Programa de Pós-Graduação em Medicina e Saúde Humana, Escola Bahiana de Medicina e Saúde Pública, Salvador, Bahia, Brazil; ^5^Department of Epidemiology, School of Public Health, Boston University, Boston, MA, United States

**Keywords:** One Health, health policy, climate change, sustainability, health promotion

## 1 Introduction

Planetary health is an inherently complex field that requires the integration of diverse expertise, ranging from environmental science and public health to economics and social sciences. Despite growing recognition of its importance and awareness, planetary health faces significant challenges in translating its goals into effective action (1). These challenges often arise from the fragmented efforts of various sectors, each with distinct priorities, methodologies, and terminologies. This fragmentation is further compounded by differences in data collection practices, funding structures, and institutional frameworks, which hinder the necessary integration for meaningful impact. To effectively address these multifaceted issues, it is necessary to adopt pragmatic approaches that emphasize achievable, impactful goals.

## 2 Discussion

A primary difficulty in establishing effective planetary health initiatives is the fragmented efforts that arise from the interdisciplinary nature of the field. Every holistic approach goes through a critical phase in which its ideals need to be materialized to have a real impact on the world (2). Despite differences in scope and focus, planetary health can draw valuable lessons from the historical evolution of the One Health movement, as this initiative similarly required the broad engagement of communities, funders, and policymakers to develop an operational framework that translated its ideals into action, showcasing the value of collaboration across disciplines (3).

Currently, different sectors, such as governmental surveillance, climate and geographical sensors, environmental science, public health, and social sciences, often operate with little communication between them (4). For instance, environmental data and health data are frequently collected and analyzed separately, complicating efforts to understand and address their interconnections. Different actors produce data that are not interoperable, hampering the development of the field. Consequently, despite the existence of valuable data, scientists often cannot leverage it to answer important questions due to the lack of integration.

The increasing specialization within scientific fields, while advancing knowledge in specific domains, often leads to a bubble where each discipline develops its own terminologies, methodologies, and paradigms (5). Although beneficial for deepening expertise within a specific field, this can hinder effective collaboration and integration necessary for addressing the multifaceted challenges of planetary health. For instance, environmental scientists may focus on metrics like biodiversity indices and carbon footprints, while public health researchers concentrate on disease incidence rates and healthcare access. Both of these fields may lack the qualitative aspect and community engagement of the social sciences. These differing priorities and approaches can lead to communication barriers and a lack of cohesive strategy.

Implementing planetary health initiatives also faces significant funding challenges, especially in low-resource settings. Despite improvements in recent years, with institutions like the Wellcome Trust supporting broad climate and health research initiatives, securing funding for interdisciplinary and innovative projects remains a persistent challenge. This is likely due to the complexity and magnitude of these projects, which require the integration of expertise, tools and human resources from different fields. Additionally, countries that fund the majority of climate research are the least affected by climate change, creating a disparity in research focus and need (6, 7). Political shifts can further complicate matters by impacting environmental and health policies, thereby affecting the continuity and stability of long-term projects (8, 9).

Brazil exemplifies both the potential and the challenges inherent in planetary health initiatives. The country has comprehensive health and climate registries and plays a central role in global climate discussions due to its vast biodiversity and significant natural resources (10, 11). Also, the country is significantly affected by extreme climate events, such as floods, wildfires, drought, escalating air pollution, amongst others (12). However, Brazil faces substantial obstacles in harnessing planetary health data effectively. The country's health and climate data are often fragmented and managed by different government institutions, which leads to issues of accessibility and integration. Bureaucratic hurdles further complicate data utilization, and disparities in the distribution of technological infrastructure between different regions worsen inequalities (13). Moreover, incomplete datasets can hinder granular analyses and the development of effective interventions.

Considering these challenges, a shift toward more pragmatic approaches in planetary health is essential. We recognize the critical need for systemic changes that align human progress with the health of the planet, as highlighted by the São Paulo Declaration on Planetary Health (14). In consonance, we support the integration of health data across sectors, the strengthening of interdisciplinary collaboration, and capacity-building initiatives that are locally tailored (Table 1). Moreover, both approaches advocate for an unified framework that enables collaboration across sectors and the construction of sustainable health infrastructure and resilient systems, with focus on early intervention and preventive measures.

**Table 1:** The table details each action and their expected impacts.

**Pragmatic action**	**Description**	**Expected impact**
Data integration	Interdisciplinary team formation: include experts from environmental science, public health, data science, social sciences, and relevant stakeholders in platform development	
	Platform revision: use a multidisciplinary approach to revise and integrate existing data platforms	Enhanced ability to identify and address interconnections between environmental changes and health outcomes; improved research capabilities and policymaking
	Guidelines development: define and share clear guidelines for data access and integration	
Capacity building	Curriculum inclusion: advocate for incorporating planetary health in formal education across relevant disciplines	
	Integrated learning: promote integrated learning sessions during graduate programs	Strengthened research capacity, increased cross-disciplinary collaboration, and enhanced ability to address complex planetary health challenges
	Workshops and Seminars: Organize regional Workshops and Seminars with community engagement components	
	Course development: develop and offer comprehensive courses and webinars on Interdisciplinary research and planetary health	
Focused research initiatives	Leverage existing capacity: utilize existing research strengths to broaden the scope without interrupting progress	
	Stakeholder consultation: gather input from key stakeholders to identify critical research areas	
	Interdisciplinary teams: include professionals from complementary fields to expand research scope	Demonstrated progress in key areas, validation of interdisciplinary approaches, and creation of scalable models for broader adoption
	Mixed methods approach: incorporate both qualitative and quantitative methods, engaging communities in the research process	
	Dissemination materials: develop executive summaries, presentations, and public materials to share research findings	
Standardization of terminologies and methodologies	Standardization task force: develop and adopt standardized terminologies and methodologies across disciplines to enhance communication and collaboration	
	Harmonization sessions: conduct workshops and meetings focused on harmonizing terminologies and methodologies	Reduced barriers to interdisciplinary collaboration, more cohesive research efforts, and clearer communication of findings
	Publication and dissemination: use high-impact journals to publish peer-reviewed guidelines, glossaries, data collection protocols, and analytical frameworks	
Integration with governmental agencies and key actors	Joint Initiatives: address local governmental institutions to design joint research and intervention initiatives that incorporate governmental input into research projects and vice versa	
	Improve transparency: government sectors should facilitate a granular understanding of the different sectors' roles and scope to enhance coordination and accountability	Enhanced relevance and implementation of research, greater public health impact, more strategic utilization of governmental resources
	Ongoing training: provide continuous training and support to government officials to ensure the integration of scientific findings into policy making	
	NGO and private sector engagement: collaborate with NGOs and private sector entities to enhance policy implementation and resource mobilization	

However, an immediate focus on targeted, practical actions is essential to making these ideals actionable. Achieving “The Great Transition” requires foundational steps that can yield measurable results in the short term, providing the groundwork for broader systemic change. Therefore, our approach complements the Declaration's vision by leveraging existing expertise to focus on achievable, impactful goals, which demonstrate the value of interdisciplinary approaches and can incentivize the broader adoption of planetary health principles. This approach involves identifying areas of weakness within health data infrastructure, building capacity where needed, and integrating experts from diverse fields to broaden the scope of initiatives without attempting to address all aspects of planetary health simultaneously.

In this context, we envision that successful initiatives will leverage existing strengths in health research and epidemiology to address specific planetary health challenges (Table 1). For example, engaging governmental surveillance sectors, integrating and analyzing large datasets from diverse sources at the inception of data curation and uncovering patterns and associations between environmental and health data are paramount. Efforts, such as the development of the One Health Index, are a manifestation of these claims, demonstrating how multisectoral indicators of human, animal, and environmental health can be integrated into a single index for comprehensive assessment and be a foundation to further actions (15).

Additionally, including communities and tailoring interventions to their unique culture, environmental exposures and genetic predispositions, are initiatives that can effectively address the links between environmental changes and climate-sensitive diseases to inform prevention and control strategies This comprehensive strategy will allow for the flourishing of the research field, the construction of a standardized language, solid and effective capacity building and integration with governmental technical areas for targeted and prioritized actions.

While the field faces significant challenges, actionable initiatives may demonstrate that targeted, strategic efforts can lead to meaningful progress. By leveraging existing expertise, fostering collaboration, and embracing innovation, it is possible to make a significant impact in the field of planetary health. Through these efforts, we hope to inspire similar initiatives globally and contribute to a more sustainable, equitable, and healthy future for all.
